# Rapid screening and identification of ACE inhibitors in snake venoms using at-line nanofractionation LC-MS

**DOI:** 10.1007/s00216-017-0531-3

**Published:** 2017-08-11

**Authors:** Marija Mladic, Tessa de Waal, Lindsey Burggraaff, Julien Slagboom, Govert W. Somsen, Wilfried M. A. Niessen, R. Manjunatha Kini, Jeroen Kool

**Affiliations:** 1 Division of BioAnalytical Chemistry, Amsterdam Institute for Molecules Medicines and Systems, Vrije Universiteit Amsterdam, De Boelelaan 1083, 1081 HV Amsterdam, The Netherlands; 2 hyphen MassSpec, Herenweg 95, 2361 EK Warmond, The Netherlands; 3 Department of Biological Science, National University of Singapore, 14 Science Drive 4, Singapore, 117543 Singapore

**Keywords:** ACE inhibitors, Snake venoms, At-line nanofractionation, Liquid chromatography-mass spectrometry

## Abstract

This study presents an analytical method for the screening of snake venoms for inhibitors of the angiotensin-converting enzyme (ACE) and a strategy for their rapid identification. The method is based on an at-line nanofractionation approach, which combines liquid chromatography (LC), mass spectrometry (MS), and pharmacology in one platform. After initial LC separation of a crude venom, a post-column flow split is introduced enabling parallel MS identification and high-resolution fractionation onto 384-well plates. The plates are subsequently freeze-dried and used in a fluorescence-based ACE activity assay to determine the ability of the nanofractions to inhibit ACE activity. Once the bioactive wells are identified, the parallel MS data reveals the masses corresponding to the activities found. Narrowing down of possible bioactive candidates is provided by comparison of bioactivity profiles after reversed-phase liquid chromatography (RPLC) and after hydrophilic interaction chromatography (HILIC) of a crude venom. Additional nanoLC-MS/MS analysis is performed on the content of the bioactive nanofractions to determine peptide sequences. The method described was optimized, evaluated, and successfully applied for screening of 30 snake venoms for the presence of ACE inhibitors. As a result, two new bioactive peptides were identified: pELWPRPHVPP in *Crotalus viridis viridis* venom with IC_50_ = 1.1 μM and pEWPPWPPRPPIPP in *Cerastes cerastes cerastes* venom with IC_50_ = 3.5 μM. The identified peptides possess a high sequence similarity to other bradykinin-potentiating peptides (BPPs), which are known ACE inhibitors found in snake venoms.

**Electronic supplementary material:**

The online version of this article (doi:10.1007/s00216-017-0531-3) contains supplementary material, which is available to authorized users.

## Introduction

Snake venoms comprise a myriad of bioactive peptides and proteins causing multiple physiological reactions upon envenomation of a prey organism. The diversity of effects involving varying mechanisms of action, together with the high target specificity of each single constituent, makes snake venoms very attractive materials in the discovery of new drugs for the treatment of different diseases. Animal venoms have been the origin of several major drugs or drug classes. One of the best known examples is provided by angiotensin-converting enzyme (ACE) inhibitors [1]. Other snake venom-derived drugs include antiplatelet agents, tirofiban [2], and eptifibatide [3], from the venoms of the saw-scaled viper, *Echis carinatus*, and the southeastern pygmy rattlesnake, *Sistrurus miliarius barbouri*, respectively.

ACE is a zinc-dependent metallopeptidase which plays an important role in regulation of blood pressure in the organism. It is responsible for the conversion of angiotensin I into angiotensin II, a very potent vasoconstrictor, and for the deactivation of bradykinin, an endogenous vasodilating peptide. Therefore, inhibition of ACE activity leads to a decrease in the blood pressure.

Discovery and development of ACE inhibitors have made a significant impact on the medical treatment of hypertension and congestive heart failure. The first ACE inhibitor registered on the market was captopril, which was synthesized based on the structure of a peptide found in 1970s in the venom of the Brazilian pit viper *Bothrops jararaca* [4–6]. Since then, a large number of peptides inhibiting ACE have been identified in snake venoms [7–9]. These peptides are named bradykinin-potentiating peptides (BPPs), owing their name to increased bradykinin activity caused by ACE inhibition. Recently published studies show that academic groups remain interested in the discovery and identification of new ACE inhibitors from animal venoms [10–13].

In general, screening, purification, and characterization of relevant bioactive compounds from complex mixtures, such as snake venoms, is a challenging and often laborious task. Many groups are successfully applying the bioassay-guided fractionation approach to identify bioactive compounds in venoms. Here, we refer to a few recent examples [13–16]. However, these studies can be very time-consuming before the bioactive compound is identified, because the bioassays are often not directly linked to the chemical identification, which is mostly performed by separately conducted MS analysis.

Recently, a new method named the at-line nanofractionation methodology, which is based on the principles of bioassay-guided fractionation, was developed and applied in screening of snake venoms for compounds affecting thrombin and factor Xa activity [17]. This method combines reversed-phase liquid chromatography (RPLC) analysis of a crude snake venom with parallel mass spectrometry (MS) detection and high-resolution nanofractionation onto 384-well plates, enabled by the presence of a post-column flow split. After nanofractionation, 384-well plates are dried to eliminate the organic modifiers present in the LC eluents and then directly bioassayed. The bioassay results are plotted in a bioactivity chromatogram resembling the bioactivity profile of a snake venom tested in the particular assay. Since the at-line nanofractionation is performed in 6-s resolution, the retention and resolution of eluting compounds from the LC separation is retained in the bioactivity chromatograms. After the bioactive peaks are identified in the bioactivity profile, the parallel MS measurement gives information on the *m*/*z* value corresponding to the bioactivity detected. Extracted ion chromatograms (XICs) of all the possible candidates are plotted and the peak shapes and retention times of the peaks are then correlated to the bioactive peaks detected in the bioactivity chromatogram [17].

In this study, the at-line nanofractionation methodology was optimized and evaluated for screening mixtures such as snake venoms towards ACE activity. The optimized method was then applied to the screening of 30 snake venoms. All snake venoms were initially screened using RPLC resulting in the identification of several snake venoms containing ACE inhibitors. Additional RPLC re-screening was performed on the snake venoms with significant positive hits to confirm the presence of the bioactive compounds. The identified bioactive peaks were correlated to corresponding accurate *m*/*z* values obtained in the parallel MS measurements. In case multiple possible *m*/*z* values were found to correlate to the bioactivity due to co-eluting compounds, a hydrophilic interaction liquid chromatography (HILIC) separation was used to re-screen the respective crude venom. The complementary HILIC separation helped narrow down the number of candidates for the bioactivity observed. After correlation of bioactivity to an accurate *m*/*z* value, the bioactive nanofractions were subsequently subjected to the nanoLC-MS/MS analysis in order to reveal the sequence of the bioactive peptides.

## Material and methods

### Chemicals

Water was purified using a Milli-Q Plus system from Millipore (Amsterdam, The Netherlands). Acetonitrile (ACN) (ULC/MS grade) and formic acid (FA) were obtained from Biosolve (Valkenswaard, The Netherlands). Tris, ZnCl_2_, glycerol, hydrochloric acid, angiotensin-converting enzyme (ACE) from rabbit lung (≥2.0 units/mg protein), and captopril were obtained from Sigma-Aldrich (Zwijndrecht, The Netherlands). *o*-Aminobenzoylglycyl-*p*-nitro-l-phenylalanyl-l-proline (Abz-Gly-*p*-nitroPhe-Pro-OH; M1100) was obtained from Bachem (Bubendorf, Switzerland). DMSO was obtained from Riedel-de Haën (Zwijndrecht, The Netherlands). Most snake venoms were obtained commercially from Kentucky Reptile Zoo (USA), Ventoxin (USA), Biotoxin (USA), Venom Supplies (Tanunda, Australia), and African Reptiles & Venoms (Johannesburg, South Africa). The *Boiga irregularis* and *Gloydius blomhoffii* venoms were kind gifts from Prof. Steve Mackessy (University of Northern Colorado, USA) and Prof. Sadaaki Iwanaga (Kyushu University, Japan), respectively. Lyophilized venoms were kept at −20 °C. Prior to analysis, snake venoms were diluted in water to an end concentration of approximately 5.0 ± 0.1 mg/mL. After analysis, the samples were kept at −80 °C for later re-analysis.

### Liquid chromatography, at-line nanofractionation, and mass spectrometry

Liquid chromatography separation was performed on a Shimadzu (‘s-Hertogenbosch, The Netherlands) UPLC system controlled via the Shimadzu Lab Solutions software. Sample injection was performed with a Shimadzu SIL-30AC autosampler using a 50-μL injection volume for RPLC separation and a 20-μL injection volume for HILIC separation. A gradient separation was performed on a Waters (Milford, MA) xBridge C_18_ column (100 × 4.6 mm; 5 μm) with an xBridge C_18_ guard column (10 × 4.6 mm; 5 μm) in RPLC mode. In HILIC mode, an Atlantis™ HILIC silica column (150 × 2.1 mm; 3 μm) was used. The columns were thermostated at 37 °C in a Shimadzu CTD-30A column oven. The solvent delivery was performed with two Shimadzu LC-30AD parallel pumps with 0.6 and 0.15 mL/min total flow rates for RPLC and HILIC separation, respectively. The mobile phases consisted of eluent A (98% H_2_O, 2% ACN, and 0.1% FA) and eluent B (98% ACN, 2% H_2_O, and 0.1% FA). The setup was optimized and validated in RPLC mode using the following gradient: a linear increase from 0 to 30% B in 20 min followed by 5-min isocratic elution at 30% B and a subsequent decrease to 0% B in 1 min with a 5-min equilibration time. The snake venoms screening for ACE activity was performed both in RPLC and HILIC mode. In the RPLC mode, venoms were analyzed using a 20-min linear gradient from 0 to 50% B followed by a linear increase to 90% B in 2 min and then a 2-min isocratic elution at 90% B before going back to 0% B in 1 min. The column was then equilibrated for 5 min. In the HILIC mode, separation started at 50% B and decreased linearly to 0% B in 20 min. This was followed by 4-min column washing at 0% B and a subsequent increase to 50% B in 1 min. The column was then equilibrated for 10 min. After the column, a flow split was introduced in a 1:9 ratio. The larger flow portion was directed to a Gilson 235P autosampler for 6-s nanofractionation onto black 384-well plates (Greiner Bio One, Alphen aan den Rijn, The Netherlands). The nanofractionated plates were subsequently dried in a Christ Rotational Vacuum Concentrator (Salm en Kipp, Breukelen, The Netherlands) RVC 2−33 CD plus and then stored at −20 °C until use. Ariadne, an in-house written software, was used to control the nanofractionation. The smaller flow portion was directed towards a Shimadzu SPD-M20A prominence diode array detector followed by a Waters Ultima quadrupole–time-of-flight (q–TOF) mass spectrometer equipped with an electrospray ionization (ESI) source. The MS was operated in positive ion mode with the following settings for the ESI source parameters: source temperature 100 °C, desolvation temperature 200 °C, capillary voltage 3 kV, and gas flow 350 L/min. Spectra were acquired at 1 spectrum/s rate in the range of *m*/*z* 50 to 2000.

### ACE bioactivity assay

ACE was stored at −20 °C as a 1-U/mL solution in 50% glycerol/50% 0.15 M Tris-HCl pH 8.3 supplemented with 5 mM ZnCl_2_. M1100 was stored as a 10 mM solution in DMSO at −20 °C. The assay was performed in a 0.15 M Tris-HCl buffer pH 8.3 supplemented with 0.5 M NaCl. Prior to pipetting, both ACE and M1100 were diluted directly in the buffer at room temperature to final concentrations of 1 mU and 40 μM, respectively. This solution was then added directly to the nanofractionated plates at room temperature using a Multidrop™ 384 Reagent Dispenser (Thermo Fisher Scientific, Ermelo, The Netherlands). The final volume of the assay was 75 μL/well. The readout of the assay was fluorescence measured with a Varioskan™ Flash Multimode Reader (Thermo Fisher Scientific, Ermelo, The Netherlands) at 320 nm excitation and 420 nm emission wavelength. The measurements were performed at 37 °C after 1 h and after 2 h from the start of addition of the assay solution to the plates. Between the two time points, the plates were incubated at 37 °C. The activity of ACE in the wells was expressed as the slope of the kinetic curve that resulted from the two time point measurements. The resulting slopes were normalized and plotted in a graph versus time of each nanofraction collected using the GraphPad Prism 6 software (La Jolla, CA, USA). The slopes were normalized by dividing each slope value with the median of all the values obtained in a single measurement.

### NanoLC-MS/MS

NanoLC separation was performed using an UltiMate 3000 RSLCnano system (Thermo Fisher Scientific, Ermelo, The Netherlands). An autosampler was run in full-loop injection mode with the injection volume set at 1 μL. The samples were separated on an analytical capillary column (150 mm × 75 μm) packed in-house with Aqua C_18_ particles (3 μm particle size and 200 Å pore diameter; Phenomenex, Utrecht, The Netherlands). The mobile phases consisted of eluent A (98% H2O, 2% ACN, 0.1% TFA) and eluent B (98% ACN, 2% H2O, 0.1% TFA). The following gradient program was used for the separation: 2-min isocratic separation at 5% B, linear increase to 80% B in 15 min, 3-min isocratic separation at 80% B, decrease to 5% B in 0.5 min, and equilibration for 9 min. The column was thermostated at 30 °C in the column compartment. Detection was carried out with a variable wavelength detector set at 254 nm followed by a micro-q–TOF mass spectrometer (Bruker, Bremen, Germany). The MS had an electrospray ionization (ESI) source and was operated in positive-ion mode. Typical settings of the ESI source parameters for the micro-q–TOF MS were the following: source temperature 200 °C, capillary voltage 4.5 kV, and gas flow 10 L/min. Spectra were acquired at 1 spectrum/s in the range of *m*/*z* 50 to 3000. MS/MS spectra were recorded in data-dependent mode using 35-eV collision energy in the CID collision cell. The Bruker DataAnalysis software was used for data analysis.

## Results and discussion

### Optimization, calibration, and evaluation of the screening method assay

The bioassay used in this study is based on the enzymatic conversion of a fluorogenic substrate, M1100 (*o*-aminobenzoylglycyl-*p*-nitro-l-phenylalanyl-l-proline; Abz-Gly-*p*-nitroPhe-Pro-OH) into a fluorescent product (Abz-Gly-OH). Incubation of known concentrations of ACE and M1100 gives a certain fluorescence that represents the basal signal of the assay. In the presence of an ACE inhibitor, a drop in the basal fluorescence is detected.

Prior to optimization of the analytical methodology and the screening of snake venoms, the ACE bioassay used in 384-well plate was optimized regarding the substrate and enzyme concentration. This was done by performing enzyme kinetic experiments using different substrate and enzyme concentrations. In total, five different enzyme concentrations (2.5, 5, 10, 20, and 40 mU) were tested in the ACE bioactivity assay, at eight substrate concentrations (5–500 μM), in a matrix fashion. For each enzyme concentration, the maximum conversion rate was reached already at 250-μM substrate concentration and it dropped down at the last substrate concentration tested. This effect was already reported for the M1100 substrate [18], and it was attributed to decreased fluorescence intensity of the product at high concentrations due to the absorption at the excitation or emission wavelength. Therefore, the results excluding the highest concentration tested (500 μM) were fitted into a Michaelis-Menten enzyme kinetic model. The *K*m value was determined to be approximately 60 μM for all enzyme concentrations tested (see Electronic Supplementary Material (ESM) Fig. S1), which is in the range of values previously reported in literature [18, 19]. The data presented was normalized for comparison reasons (ESM Fig. S1). The lowest enzyme concentration (2.5 mU) showed a lower signal-to-noise ratio (S/N) (larger standard error bars), while for the higher concentrations, no significant difference could be seen. Therefore, a 5-mU concentration of the enzyme was considered as optimal for further method optimization and validation. The optimal substrate concentration was considered to be 60 μM, corresponding to its *K*m value. However, for economic reasons, the concentration of both the enzyme and the substrate were lowered to 1 mU and 40 μM, respectively. The lowering of enzyme and substrate concentration slowed down the enzymatic reaction for a large percentage compared to the conditions determined to be optimal at first. However, when using sufficiently long incubation times, low enzyme concentrations will yield a sufficient assay window for obtaining good S/N, also in combination with much lower than the *K*m substrate concentrations. In practice, these assay conditions allowed for two measurement points at 1 and 2 h after the beginning of the incubation resulting in repeatable slope determination of the enzymatic product formation.

Optimization was followed by transfer of the bioassay to the nanofractionation setup for calibration and evaluation in which parallel LC–MS analysis and nanofractionation is performed in the same setup as previously described [17]. Calibration and validation of the screening method was done in the RPLC mode. For this, different concentrations of captopril (5 nM to 20 μM) were injected (50 μL) onto the LC column and nanofractionated in serpentine fashion onto 384-well plates using a 6-s resolution. The plates containing nanofractions were then dried and subsequently bioassayed. For each well, a time point was assigned and a slope of the enzymatic reaction was calculated and plotted versus time. The time point of each well was defined as half the fractionation resolution time plus the fractionation resolution multiplied by the number of preceding fractions. That means that the first well corresponded to the 0.05 min time point and each next well corresponded to the time point that is 0.1 min (6 s) increased compared to the previous one.

**Fig. 1:**
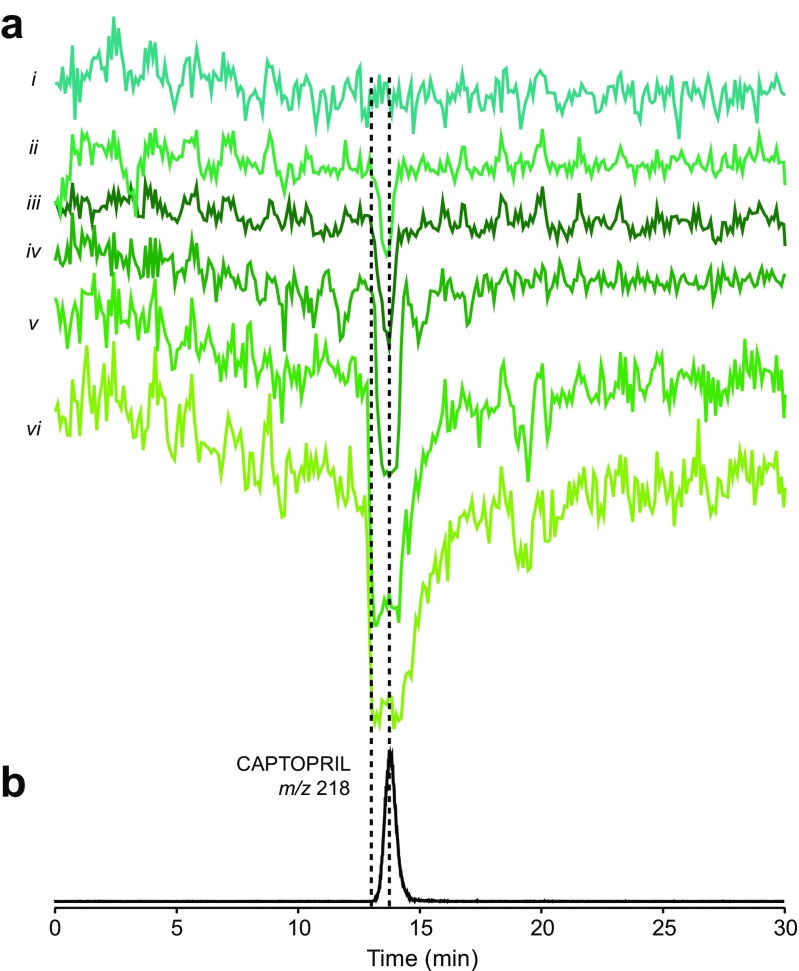
Calibration and evaluation of the at-line nanofractionation methodology for screening complex mixtures for ACE inhibitors. **a** *Traces i*–*vi* represent reconstructed bioactivity chromatograms that resulted from the bioassay after 50-μL injection of different concentrations of captopril (5 nM to 200 μM). Fractions were collected in 6-s resolution. **b** The extracted ion current of captopril is given for correlation between the MS and bioassay data

The results obtained from the bioassay performed after nanofractionation of different captopril concentrations are presented in Fig. 1 as bioactivity chromatograms. Each point in the bioactivity chromatogram represents the slope of the enzymatic reaction of the corresponding well on the 384-well plate. The limit of detection was found to be at the 50-μL injection of 40-nM captopril (2 pmols/injection), a concentration which is about four times higher than the IC_50_ value of captopril. Therefore, the method developed is suitable for detecting relatively low concentrations of potent ACE inhibitors (low IC_50_ values), while the compounds with lower potency (high IC_50_ values) need to be present in relatively high concentrations in order to be detected.

### Screening of snake venoms

In total, the venoms of 30 different species were screened with the optimized bioassay conditions in RPLC mode. A semi-quantitative estimation of the ACE inhibitory activity in 30 snake venoms that were screened (for details, see bioactivity chromatograms in Fig. S2 in the ESM) is given in Table 1. The venoms that showed a significant presence of ACE inhibitors are indicated with a “+++” sign, the venoms that showed moderate inhibition are indicated with a “++” sign, the venoms that showed little inhibition are indicated with a “+” sign, while the venoms that showed no inhibition are marked with a “−” sign. It is important to note that the detection of (significant) inhibitory activity depends on various factors. Its absence does not necessarily mean that the specific venom analyzed does not contain the ACE inhibitory peptides such as BPPs. The detection of the inhibitory peptides, under the experimental conditions used in this study, is dependent on the concentration of the peptide and its potency. Peptides with high potency can be easily detected in relatively low concentrations, while the peptides with low potencies will be detected only when they are present in relatively high concentrations. Moreover, the detection of ACE inhibitors in the venom may be influenced by the presence of other compounds in the venom, in particular the presence of snake venom proteases that could cleave the peptide-based substrate and elevate the basal signal in the bioactivity chromatogram, and mask the presence of inhibitors. Such proteases were detected in most of the venoms used in this study (Fig. S2 in the ESM).

**Table 1:** List of snake species screened towards ACE inhibition

	Snake species	Inhibitory activity
1	*Agkistrodon contortrix contortrix*	++
2	*Agkistrodon contortrix laticinctus*	+
3	*Agkistrodon contortrix mokasen*	+
4	*Agkistrodon bilineatus*	−
5	*Agkistrodon piscivorus conanti*	+
6	*Agkistrodon piscivorus piscivorus*	+
7	*Atropoides mexicanus*	++
8	*Atheris squamigera*	++
9	*Bitis arietans*	−
10	*Boiga irregularis*	−
11	*Bothrops alternatus*	−
12	*Bothrops atrox*	+
13	*Bungarus candidus*	−
14	*Calloselasma rhodostoma*	+
15	*Cerastes cerastes cerastes*	+++
16	*Crotalus adamanteus*	+++
17	*Crotalus atrox*	−
18	*Crotalus basiliscus*	−
19	*Crotalus culminatus*	−
20	*Crotalus durissus cumanensis*	++
21	*Crotalus durissus terrificus*	−
22	*Crotalus vergrandis*	−
23	*Crotalus viridis viridis*	+++
24	*Gloydius blomhoffii*	−
25	*Hemachatus haemachatus*	−
26	*Naja nigricollis*	−
27	*Oxyuranus microlepidotus*	−
28	*Oxyuranus scutellatus*	−
29	*Pseudonaja affinis*	−
30	*Pseudonaja nuchalis*	−

The venoms that showed a significant presence of ACE inhibitors are indicated with a “+++” sign, the venoms that showed moderate inhibition are indicated with a “++” sign, the venoms that showed little inhibition are indicated with a “+” sign, while the venoms that showed no inhibition are marked with a “−” sign. The inhibitory activity was evaluated in the at-line nanofractionation approach after injection of 250 μg venom

A significant ACE inhibition was observed in the venoms of three species, i.e., the Saharan horned viper, *Cerastes cerastes cerastes*, the eastern diamondback rattlesnake, *Crotalus adamanteus*, and prairie rattlesnake, *Crotalus viridis viridis*, so these three venoms were selected for the full identification of the bioactive compounds.

### Identification of bioactive peptides

The steps taken to go from measured inhibitory bioactivity, i.e., a negative peak in the bioactivity chromatogram, to the identification of inhibitory peptides are described on the example of screening the snake venom from *C. cerastes cerastes*.

**Fig. 2:**
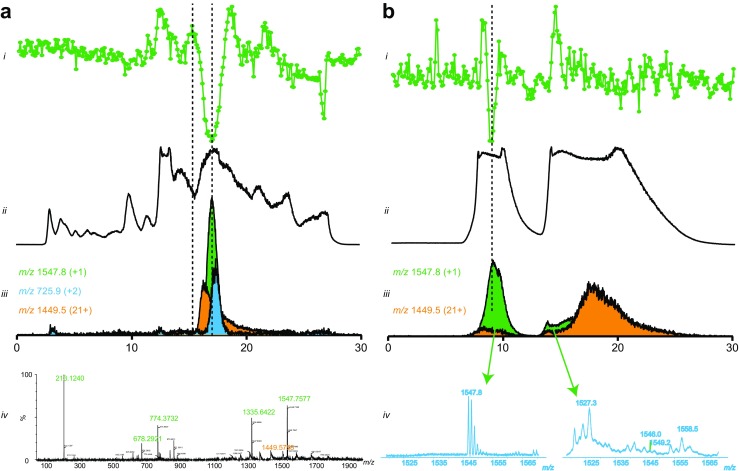
Screening of the venom from the horned desert viper (*Cerastes cerastes cerastes*) for ACE inhibitors. **a** The results of the screening performed after the RPLC separation. **b** The results of the screening preformed after the HILIC separation. *i* Reconstructed bioactivity chromatograms after corresponding LC separation and nanofractionation of a crude venom. Fractions were collected with 6-s resolution onto 384-well plates after 50-μL (RPLC separation) and 20-μL (HILIC separation) injection of the crude venom. *ii* Total ion current (TIC) and *iii* extracted ion currents (XICs) of the potential bioactive compounds obtained from the corresponding MS measurement. *iv* MS spectra corresponding to the bioactivity detected

The results of the initial screening of *C. cerastes cerastes* venom, performed with RPLC separation, are presented in Fig. 2a. One inhibitory peak is detected in the bioactivity chromatogram at the retention time of 15.95 min. In the corresponding MS spectrum, multiple *m*/*z* values are found that could correspond to the bioactivity. However, after plotting the XICs for these *m*/*z* values, only the trace for *m*/*z* 1547 corresponded to the bioactivity peak in both peak shape and retention time, leading to the conclusion that the bioactive is most likely a peptide with a mass of 1546 Da. The same snake venom was then re-screened for confirmation of the bioactivity. For this, bioactivity screens were performed after the separation in both RPLC and HILIC mode. Re-screening after RPLC separation gave the same results (data not shown). The results of the screening after the HILIC separation are presented in Fig. 2b. Only one inhibition peak was detected. As expected, the negative peak in HILIC is lower than in RPLC since the injection volume used was 20 μL for HILIC and 50 μL for RPLC. After plotting the XICs for the *m*/*z* values that could correspond to the bioactivity, again only the trace for *m*/*z* 1547 matched the bioactivity peak with respect to peak shape and retention time.

**Fig. 3:**
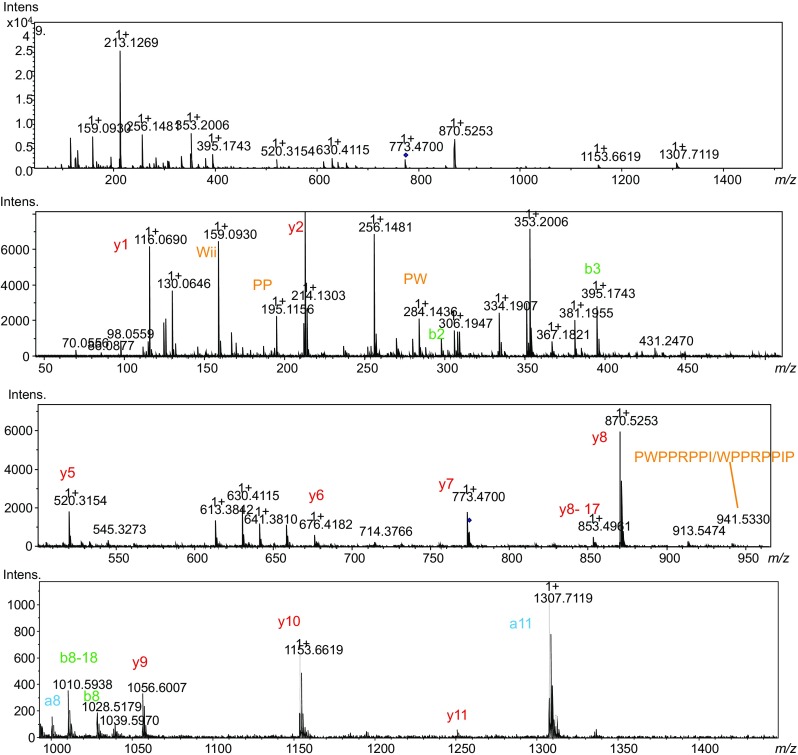
Annotated fragmentation spectra of ACE inhibitor with *m*/*z* 1547.626 found in the venom of the Saharan horned viper (*C. cerastes cerastes*)

After identifying the *m*/*z* and consequently the molecular mass of the potential bioactive peptide, i.e., 1546 Da, the content of one of the wells containing the bioactive peptide was injected directly and analyzed using nanoLC-MS/MS. Analysis of the MS spectrum and fragmentation spectra resulted in full sequence identification (Fig. 3). After analysis of the fragmentation spectra, the following sequence for the bioactive peptide was derived pEWPPWPPRPP(I/L)PP, where pE represents pyroglutamate. The proposed structure shows similarity with BPPs found in other snake venoms that usually contain a PP sequence at the C-terminal and pyroglutamate at the N-terminal of the peptide [8, 20]. Based on the analogy with other BPPs, the presence of Ile rather than Leu could be assumed, but actual differentiation between Ile and Leu was not possible under the experimental conditions used.

**Fig. 4:**
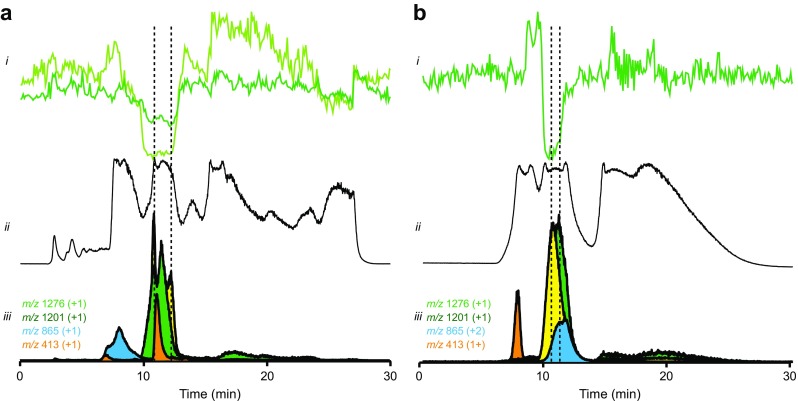
Screening of venom from the eastern diamondback rattlesnake (*Crotalus adamanteus*) for ACE inhibitors. **a** The results of the screening performed after the RPLC separation. **b** The results of the screening preformed after the HILIC separation. *i* Reconstructed bioactivity chromatograms after corresponding LC separation and nanofractionation of a crude venom. Fractions were collected with 6-s resolution onto 384-well plates after 50-μL (RPLC separation) and 20-μL (HILIC separation) injection of the crude venom. *ii* Total ion current (TIC) and *iii* extracted ion currents (XICs) of the potential bioactive compounds obtained from the corresponding MS measurement

**Fig. 5:**
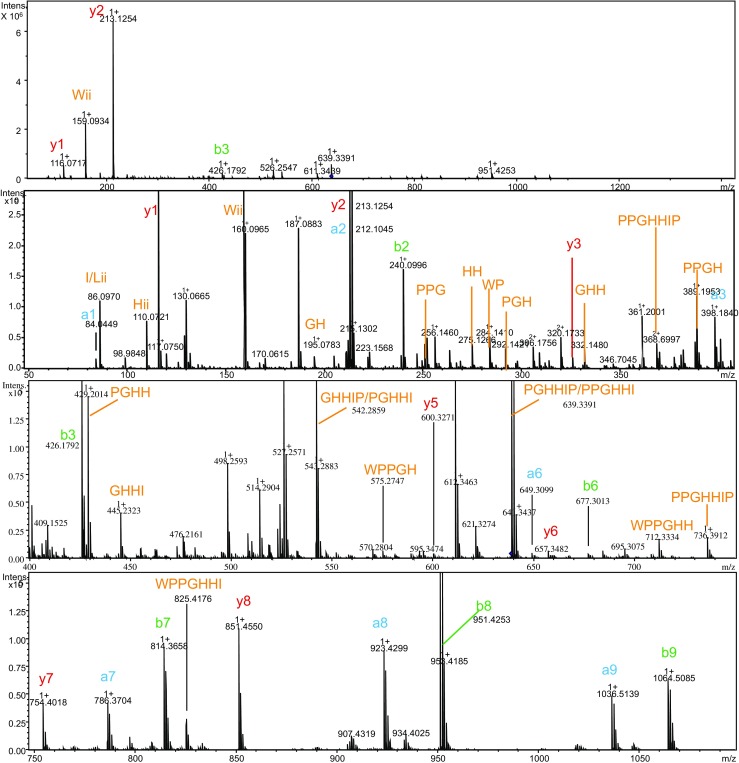
Annotated fragmentation spectra of ACE inhibitor with *m*/*z* 1276.626 found in the venom of the eastern diamondback rattlesnake (*C. adamanteus*)

**Fig. 6:**
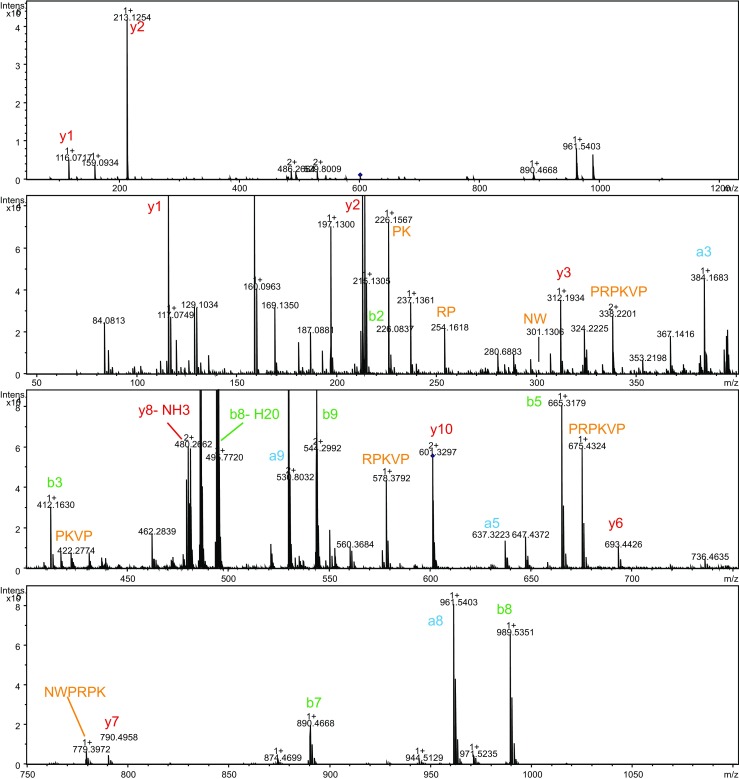
Annotated fragmentation spectra of ACE inhibitor with *m*/*z* 1201.652 found in the venom of the eastern diamondback rattlesnake (*C. adamanteus*)

The same approach was used to analyze the ACE activity profile of snake venom from *C. adamanteus*. The results of the screening are shown in Fig. 4. Two bioactivity peaks were identified in the bioactivity profiles after both the RPLC (Fig. 4a) and the HILIC separation (Fig. 4b) and they were found to correspond to *m*/*z* values 1276 and 1201. The nanoLC-MS/MS analysis of the bioactive wells and sequencing of these bioactive peptides resulted in the following sequences: pEQWPPGHH(I/L)PP and pENWPRPKVPP corresponding to *m*/*z* 1276.6264 and 1201.6520, respectively. Annotated MS/MS spectra can be found in Figs. 5 and 6 accompanied with the *m/z* values identified and their corresponding mass accuracy shown in Tables S1 and S2 in the ESM, respectively. The two ACE inhibitory peptides were found in the *C. adamanteus* venom in the previous study [9]. The first peptide (pEQWPPGHH(I/L)PP) described here is identical with this previous study [9], while the second peptide (pENWPRPKVPP) differed a few amino acid residues. The sequence determined in our study is strongly supported by the MS/MS data; most of the b and y ions were detected together with the internal sequence ions supporting our interpretation (see Fig. 6), and the mass accuracy of all the ions detected was within 7 ppm (see Table S2 in the ESM). Further, the accurate mass measured (1201.652) is closer to the theoretical mass of the sequence proposed in this study (1201.648) compared to the theoretical mass of the sequence previously proposed by Wermelinger et al. (1201.612). Our proposed sequence is also strongly supported by Eichberg et al. [21], who reported the sequences for the same two peptides, and the *C. adamanteus* transcriptome described by Rokyta et al. [22]. They proposed the presence of two potential BPPs, QNWPRPKVPP and QQWPPGHHIPP, in the Uniprot entry: J3S3U4).

Here, it should be noted that the sequence described by Wermelinger et al. [9] could also be derived due to a slightly different interpretation of the MS/MS spectra. The fragments due to internal sequence ions greatly assisted in deriving the correct sequence from the MS/MS spectra in our study of this particular peptide. However, it stays unclear whether the alternative sequence (pEGGWPRNPIPP) derived by Wermelinger et al. exists in *C. adamanteus* venoms from other regions or it was derived due to low accuracy measurements (133 ppm) and/or different interpretation of the MS/MS spectra.

Finally, the ACE activity profile for the venom from prairie rattlesnake, *C. viridis viridis*, revealed two major bioactive compounds present in the venoms with the *m*/*z* values 1209 and 1276 (ESM, Fig. S3). The peptide with *m*/*z* 1276 had the same sequence as the BPP with the same *m*/*z* value that was found in *C. adamanteus*, while the peptide with *m*/*z* 1209 was a new peptide with the following sequence pE(I/L)WPRPHVPP. For detailed information on the bioactivity data, the extracted ion currents of the bioactive compounds, and fragmentation table of fragment ions corresponding to the peptide pE(I/L)WPRPHVPP, please see Fig. S3 and Table S3 in the ESM.

### Sequence confirmation and bioactivity determination of newly identified BPPs

Full sequence and the bioactivity of new bioactive peptides was determined. For this purpose, two possible L/I variants were synthesized for both peptides and tested for their chromatographic behavior. The synthetic peptides showed slightly different chromatographic behavior, which was enough to identify the bioactive peptide after comparing peak shapes and retention times of synthesized peptides with peak shape and retention time of the bioactive peptide from the crude venom analysis. Based on the LC-MS analyses, sequences of the bioactive compounds were found to be pELWPRPHVPP (*m*/*z* 1209) for the peptide found in *C. viridis viridis* venom and pEWPPWPPRPPIPP (*m*/*z* 1547) for the peptide found in *C. cerastes cerastes* venom.

The dose-response curves of all four synthetic peptides were determined and the IC_50_ values for each peptide were determined in the ACE bioactivity assay. The IC_50_ values (Fig. S4 in the ESM) of the peptides were found to be in low micromolar range: 1.09 μM for pELWPRPHVPP, 1.01 μM for pEIWPRPHVPP, 3.47 μM for pEWPPWPPRPPIPP, and 3.37 μM for pEWPPWPPRPPLPP. Notably, peptide variants differing only in L/I showed similar bioactivity suggesting that the exchange between these two conserved, aliphatic amino acid residues does not significantly influence the bioactivity of these peptides.

## Conclusion

A total of 30 snake venoms were screened for the presence of ACE inhibitors using at-line nanofractionation approach. Five major bioactive peaks in the venoms of three different species were detected using the newly developed method. The high-resolution at-line nanofractionation allowed to keep the resolution of LC separation and thereby allowed to accurately correlate peak shape and retention time of negative bioactive peaks observed in the bioactivity chromatograms with the XICs from the parallelly obtained MS data. A complementary HILIC separation of a crude snake venom was introduced to successfully narrow down the number of candidate *m*/*z* values for the bioactive peptides. The (partial) peptide sequence was determined by direct analysis of a bioactive well using accurate mass nanoLC–MS/MS. The developed methodology is therefore an excellent tool for rapid screening of snake venoms for ACE inhibitors followed by straightforward identification of the hits found. Furthermore, the use of this method can be extended to other drug targets and natural sources.

## Electronic supplementary material

ESM 1 (PDF 852 kb)
