# Supplementary material for: Rapid screening and identification of ACE inhibitors in snake venoms using at-line nanofractionation LC-MS
Source: Anal Bioanal Chem. 2017 Aug 11;409(25):5987–97. doi: 10.1007/s00216-017-0531-3 (PMC5602078; doi:10.1007/s00216-017-0531-3)
Supplement: Supplementary file 1 — (PDF 852 kb) [file 216_2017_531_MOESM1_ESM.pdf]

Analytical and Bioanalytical Chemistry

Electronic Supplementary Material

Rapid screening and identification of ACE inhibitors in snake venoms using at-line nanofractionation LC-MS

Marija Mladic, Tessa de Waal, Lindsey Burggraaff, Julien Slagboom, Govert W. Somsen, Wilfried M.A. Niessen, R. Manjunatha Kini, Jeroen Kool

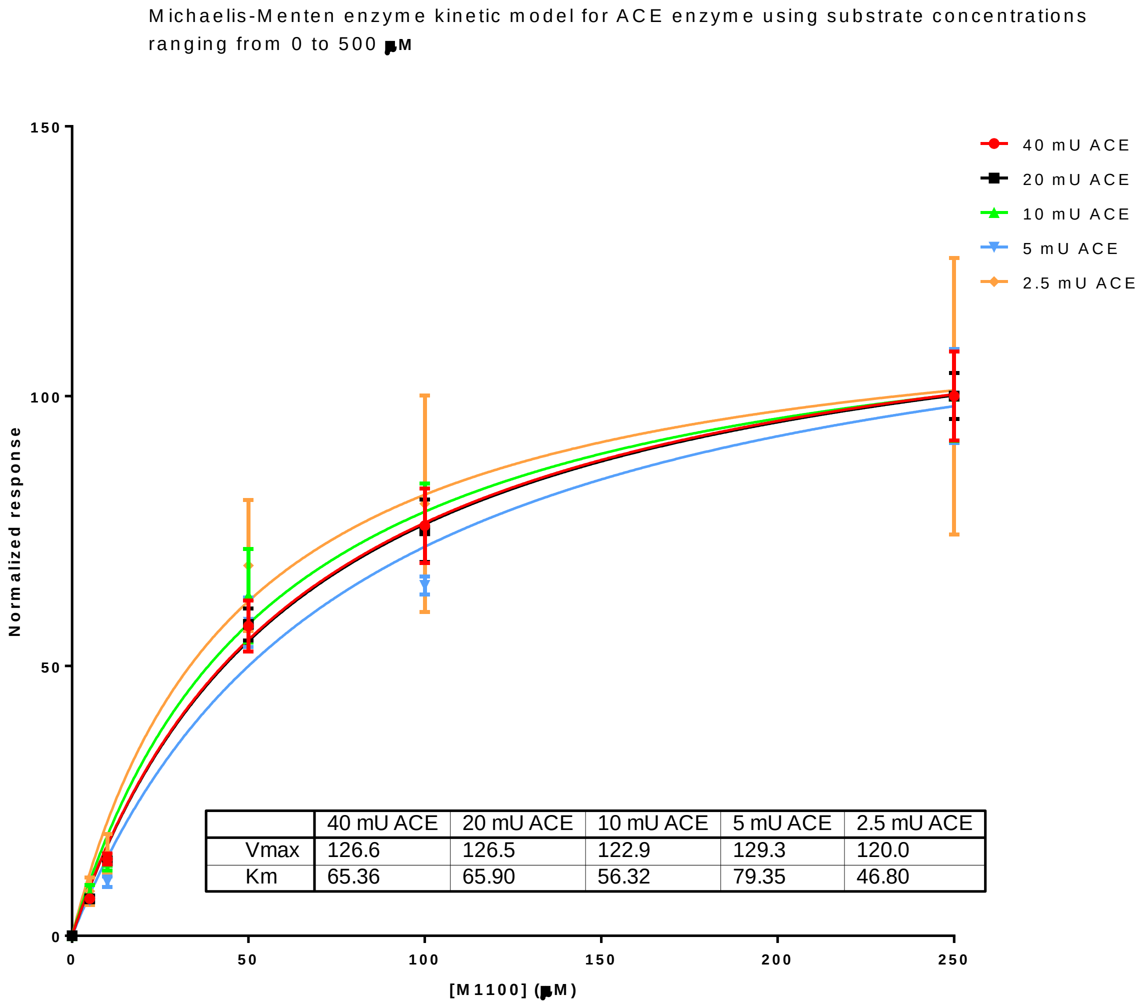

Fig. S1 Michaelis-Menten enzyme kinetic model for ACE enzyme using substrate concentrations ranging from 0 to 500 µM

Fig S2 Bioactivity profiles of venoms from 30 snake (Table 1) species screened for their inhibitory activity against ACE

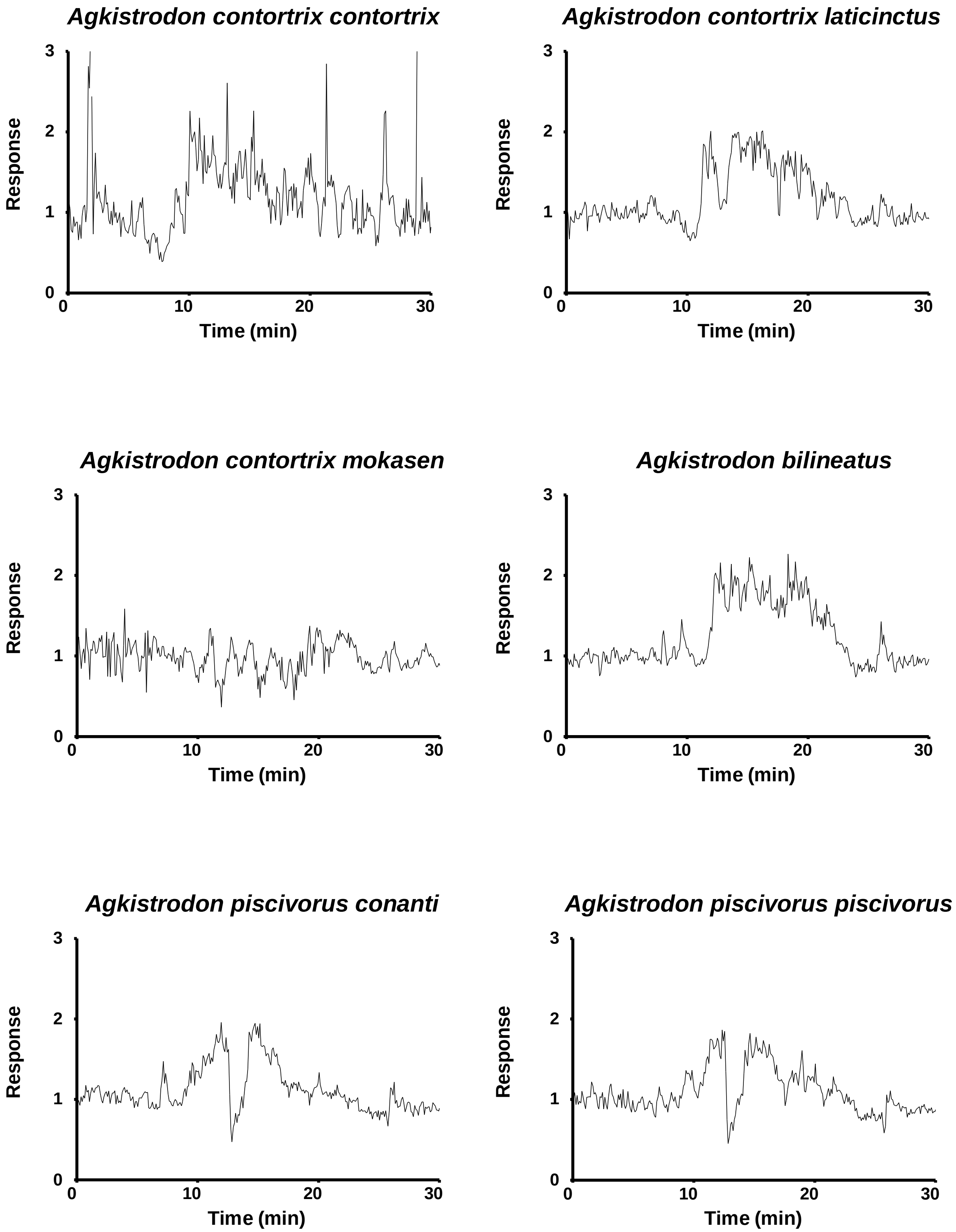

Fig S2 Bioactivity profiles of venoms from 30 snake (Table 1) species screened for their inhibitory activity against ACE (continued)

Atheris squamigera

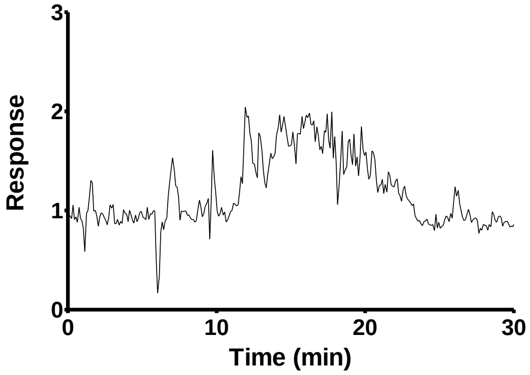

Atropoides mexicanus

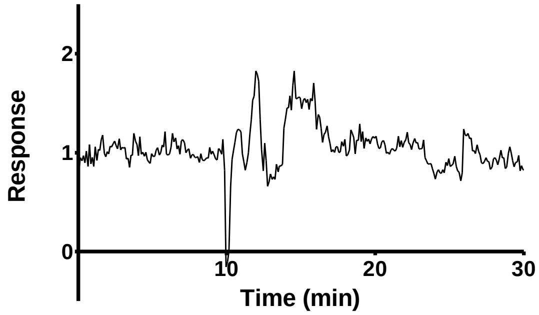

Bitis arietans

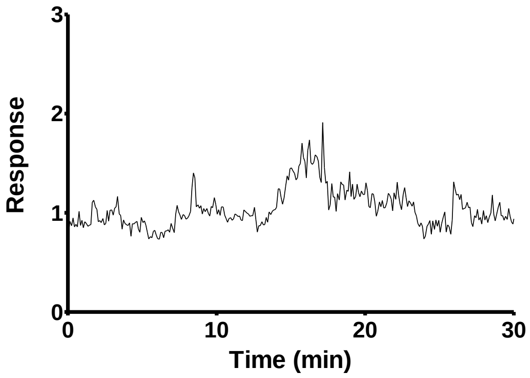

Boiga irregularis

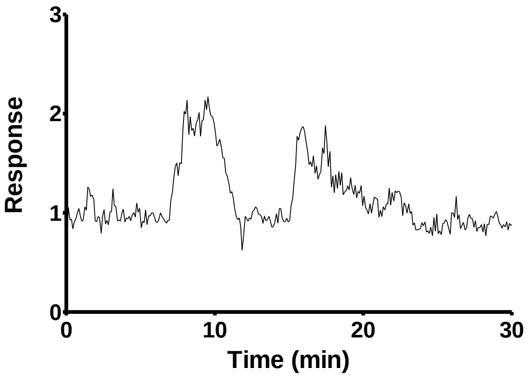

Bothrops alternatus

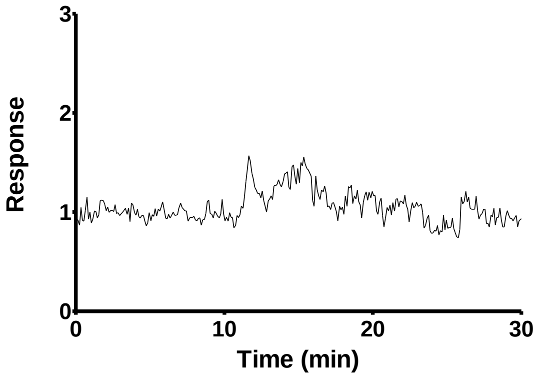

Bothrops atrox

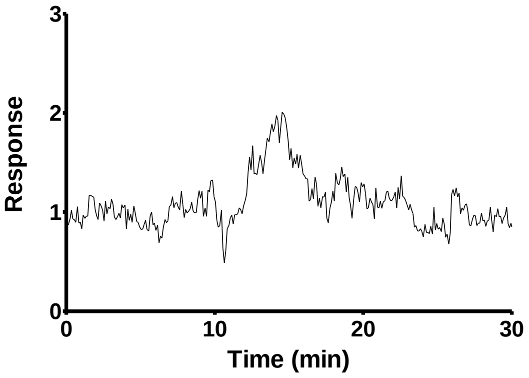

Fig S2 Bioactivity profiles of venoms from 30 snake (Table 1) species screened for their inhibitory activity against ACE (continued)

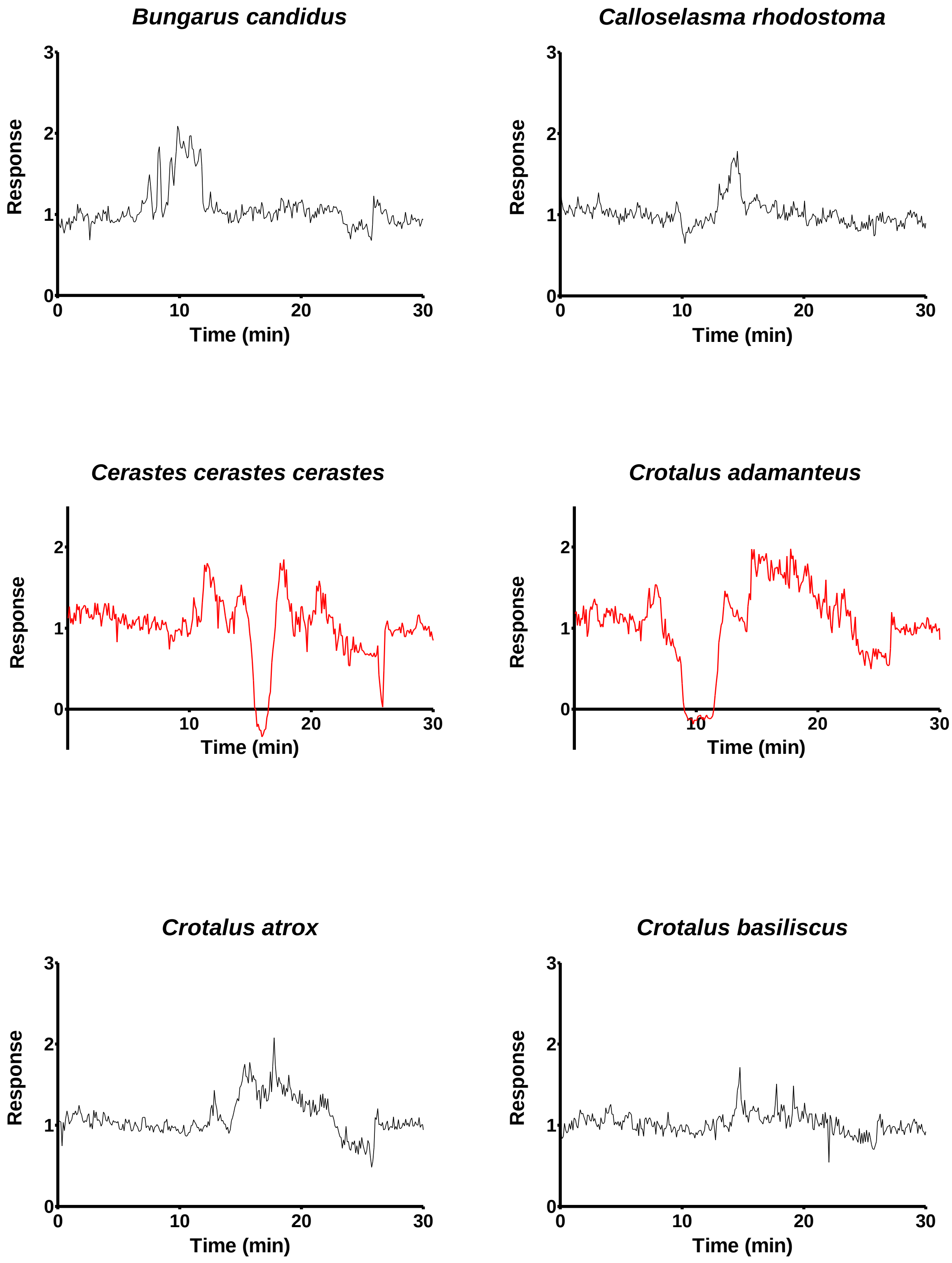

Fig S2 Bioactivity profiles of venoms from 30 snake (Table 1) species screened for their inhibitory activity against ACE (continued)

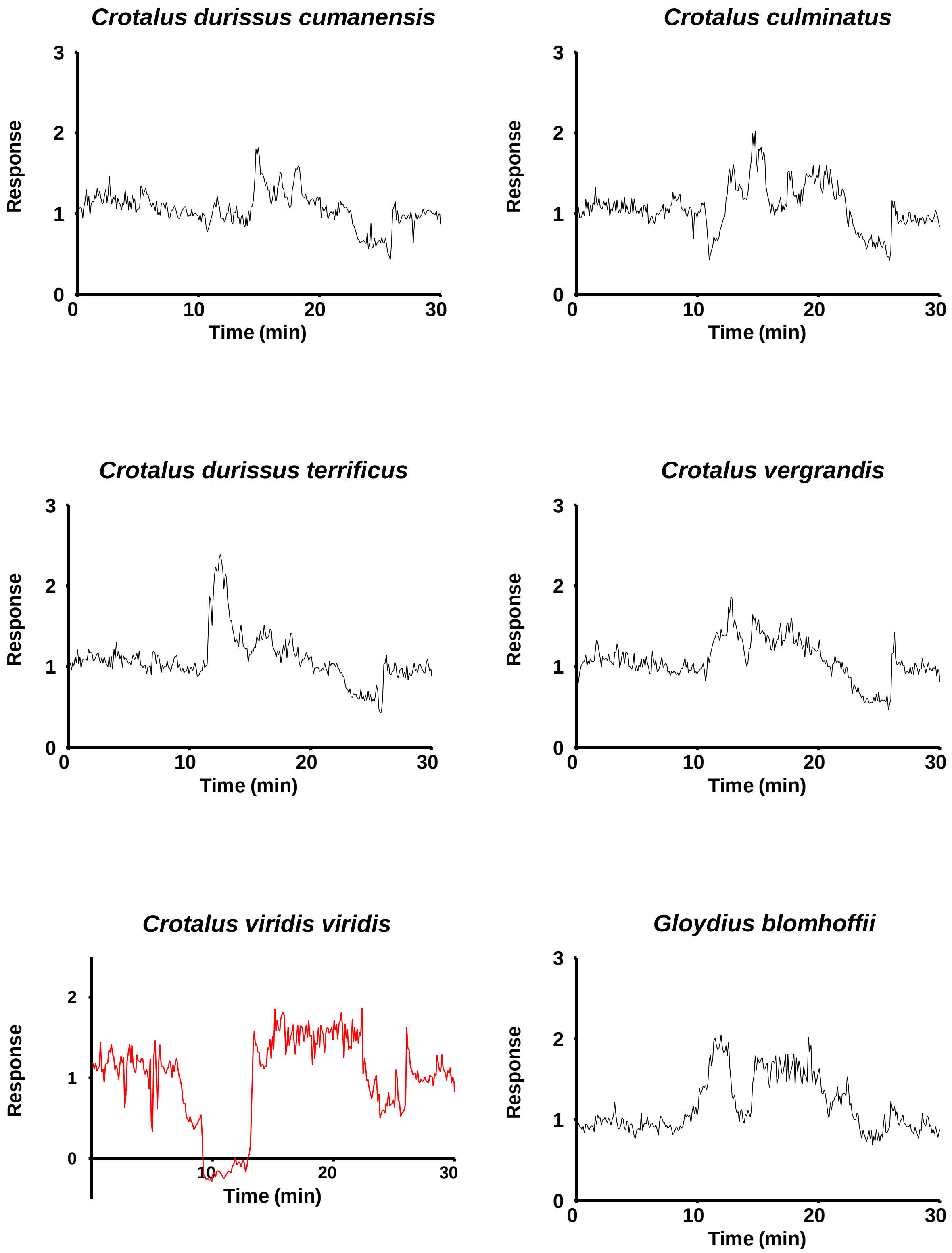

Fig S2 Bioactivity profiles of venoms from 30 snake (Table 1) species screened for their inhibitory activity against ACE (continued)

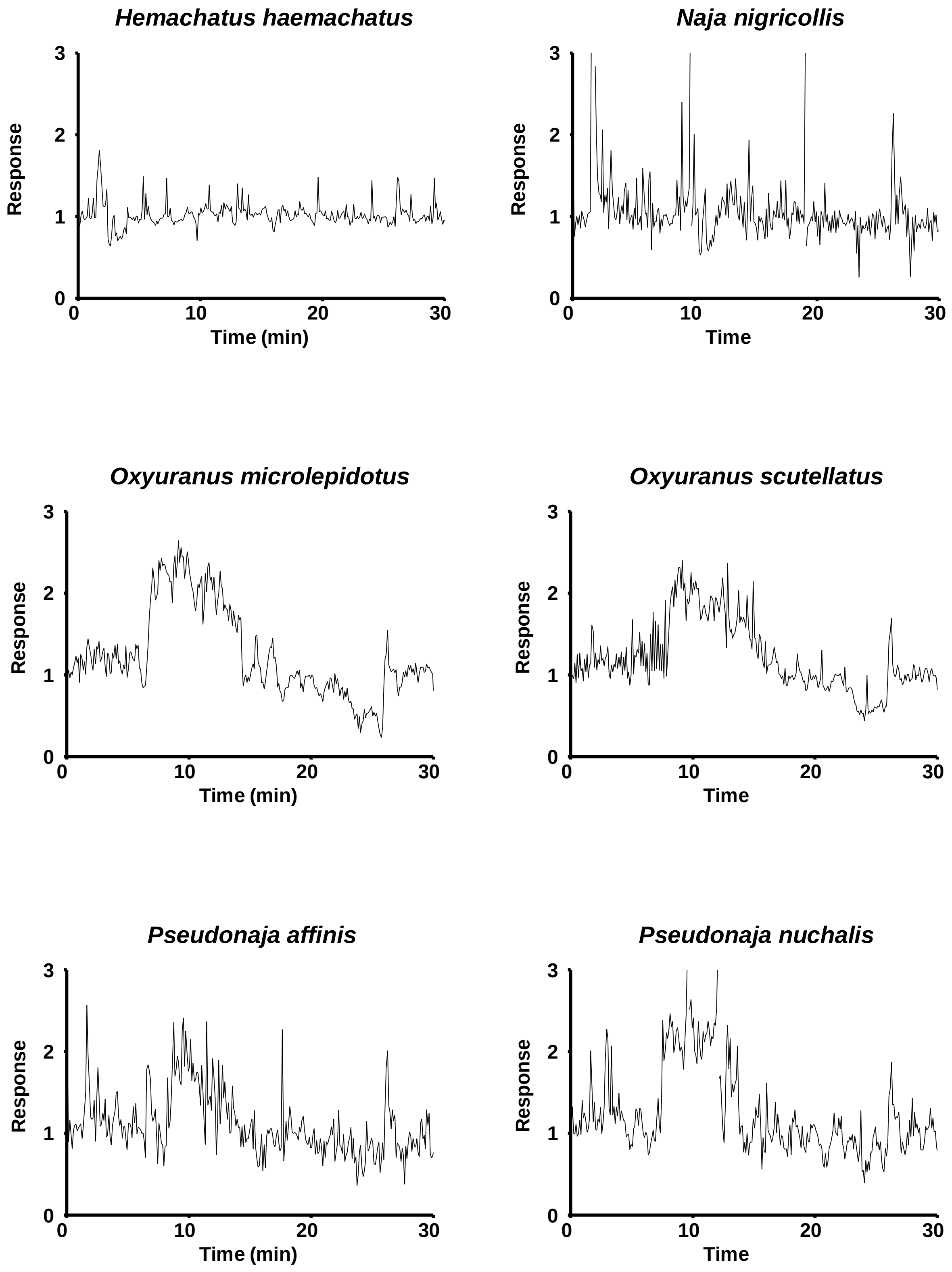

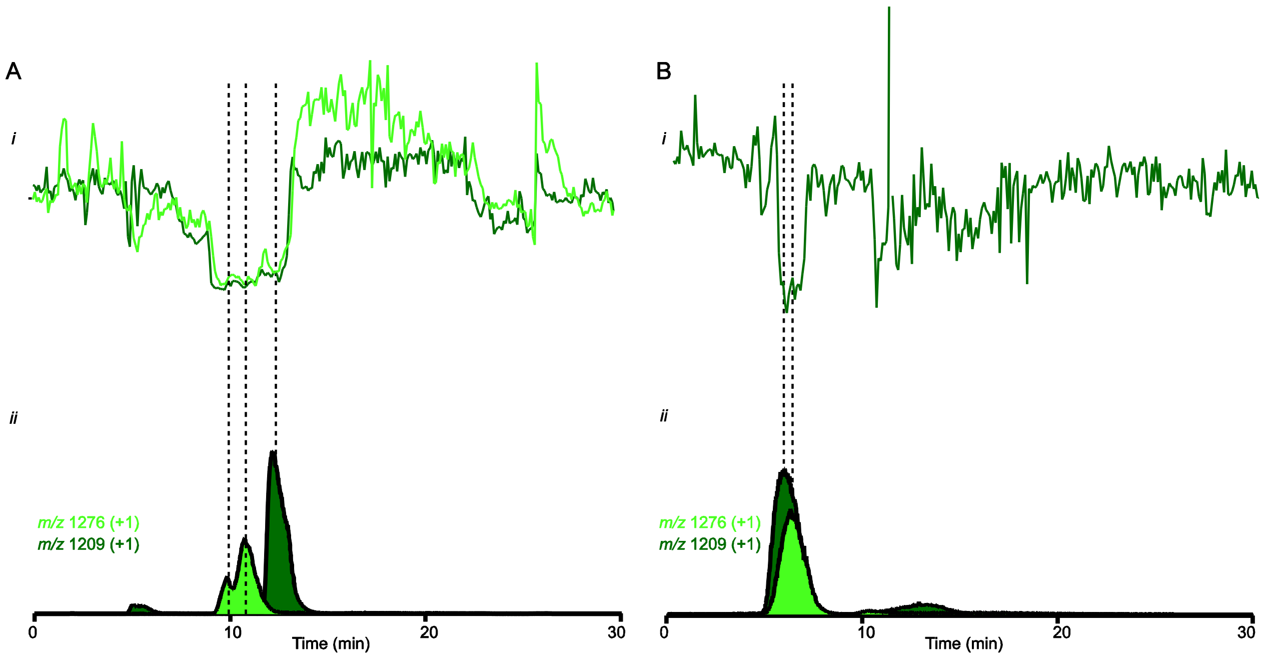

Fig. S3 Screening of venom of the prairie rattlesnake (*Crotalus viridis viridis*) for ACE inhibitors. A) The results of the screening performed after the RPLC separation. B) The results of the screening preformed after the HILIC separation. i Reconstructed bioactivity chromatograms after corresponding LC separation and nanofractionation of a crude venom. Fractions were collected with 6-s resolution onto 384-well plates after 50-µL (RPLC separation) and 20-µL (HILIC separation) injection of the crude venom. ii Extracted ion currents (XICs) of the potential bioactives obtained from the corresponding MS measurement.

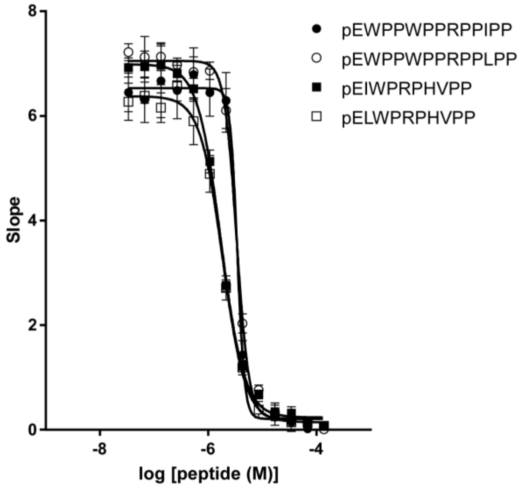

Fig. S4 Concentration-response curves of four synthesized peptides measured in the ACE activity assay. The serial dilutions of the peptides were tested in the assay using 1 mU/mL ACE enzyme and 40 µM substrate concentration. Obtained IC_{50} values for the peptides were: 3.47 µM for pEWPPWPPRPPIPP (filled circles), 3.37 µM for pEWPPWPPRPPLPP (open circles), 1.01 µM for pEIWPRPHVPP (filled squares), and 1.09 µM for pELWPRPHVPP (open squares).

Table S1 Mass accuracy of ions detected in the fragmentation spectra (Fig. 5) of peptide with *m/z* 1276. 626 found in venom of *Crotalus adamanteus*. The mass error is calculated as measured *m/z* – calculated *m/z* (in mDa)

Proposed interpretation of the ion detected	Measured accurate *m/z*	Calculated exact *m/z*	Mass error (mDa)
a3	398.1840	398.1829	+1.1
a6	649.3099	649.3099	0
a7	786.3704	786.3688	+1.6
a8	923.4299	923.4277	+2.2
a9	1036.5140	1036.5120	+2.2
b2	240.0996	240.0985	+1.1
b3	426.1792	426.1778	+1.4
b4	523.2316	523.2305	+1.1
b6	677.3013	677.3048	–3.5
b7	814.3658	814.3637	+2.1
b8	951.4253	951.4226	+2.7
b9	1064.5090	1064.5007	+1.8
y11	1276.6260	1276.6230	+3.6
y8	851.4550	851.4528	+2.2
y7	754.4018	754.4000	+1.79
y6	657.3482	657.3473	+0.9
y5	600.3271	600.3258	+1.3
y4	463.2678	463.2669	+0.9
y3	326.2090	326.2080	+1
y2	213.1254	213.1240	+1.4
y1	116.0717	116.0712	+0.5
PPGHHIP	736.3912	736.3895	+1.7
WPPGHHI	825.4176	825.4161	+1.5
PGHHIP/PPGHHI	639.3391	639.3367	+2.4
WPPGHH	712.3334	712.3320	+1.4
GHHIP/PGHHI	542.2859	542.2840	+1.9
WPPGH	575.2747	575.2731	+1.61
GHHI	445.2323	445.2312	+1.1
PGHH	429.2014	429.1999	+1.5
PPGH	389.1953	389.1938	+1.5
HHI	388.2108	388.2098	+1
GHH	332.1480	332.1472	+0.8
PGH	292.1421	292.1410	+1.1
PPG	252.1359	252.1349	+1
HHI	275.1266	275.1257	+0.9
WP	284.1410	284.1399	+1.1
IP	211.1455	211.1447	+0.8

Table S2 Mass accuracy of ions detected in the fragmentation spectra (Fig. 6) of peptide with *m/z* 1201. 652 found in venom of *Crotalus adamanteus.* The mass error is calculated as measured *m/z* – calculated *m/z* (in mDa)

Proposed interpretation of the ion detected	Measured accurate *m/z*	Calculated exact *m/z*	Mass error (mDa)
a3	384.1683	384.1672	+1.1
a5	637.3223	637.3211	+1.2
a8	961.5403	961.5372	+3.1
a9 2+	529.8009	529.7989	+2
a9	1058.5940	1058.5900	+4
b2	226.0837	226.0828	+0.9
b3	412.1630	412.1621	+0.9
b5	665.3179	665.3160	+1.9
b7	890.4637	890.4637	0
b8	989.5351	989.5321	+3
$b9^{2+}$	543.7981	543.7964	+1.7
y1	116.0717	116.0712	+0.5
y2	213.1254	213.1240	+1.4
y3	312.1934	312.1924	+1
y6	657.3482	657.3473	+0.9
y7	754.4018	754.4000	+1.79
y8	851.4550	851.4528	+2.2
y7	754.4018	754.4000	+1.79
$y10^{2+}$	601.3297	601.3280	+1.7
y10	1201.6520	1201.6482	+3.8
y8-17.026548	959.5246	959.5467	–22.1
$y8\text{-}17.026548^{2+}$	480.2662	480.2773	–11.1
b8-18.010565	971.5249	971.5215	+3.4
$b8\text{-}18.010565^{2+}$	486.2664	486.2647	+1.7
WPRPKV	764.4586	764.4872	–28.6
NWPRPK	779.3972	779.4317	–34.5
RPKVP/PRPKV	578.3792	578.3779	+1.3
RPKVP/PRPKV -28	550.3842	550.3830	+1.2
PKVP	422.2774	422.2768	+0.6
PRPK	479.3107	479.3095	+1.2
KVP/PKV	325.2250	325.2240	+1
RPK	382.2575	382.2567	+0.8
VP	197.1300	197.1290	+1
PK	226.1567	226.1556	+1.1
RP	254.1618	254.1617	+0.1
NW	301.1306	301.1301	+0.5

Table S3 Mass accuracy of ions detected in the fragmentation spectra of peptide with *m/z* 1209.655 found in venom of *Crotalus viridis viridis.* The mass error is calculated as measured *m/z* – calculated *m/z* (in mDa)

Proposed interpretation of the ion detected	Measured accurate *m/z*	Calculated exact *m/z*	Mass error (mDa)
a2	197.1296	197.129	0.6
a3	383.2077	383.2084	0.7
a7	870.4753	870.4739	1.4
a8	969.5464	969.5423	4.08
b2	225.1245	225.124	0.5
b3	411.2052	411.2033	1.9
b5	664.3595	664.3571	2.4
b6	761.4155	761.4099	5.6
b7	898.4726	898.4688	3.8
b8	997.5417	997.5372	4.46
y10	1209.655	1209.653	1.2
y7	799.4607	799.4579	2.8
y3	312.1932	312.1923	0.9
y2	213.128	213.124	4
y1	116.0687	116.0712	2.5
WRPPHVP	870.4753	870.4739	1.4
WRPPHV -28	745.4273	745.4262	1.1
RPPHV -28	559.3495	559.3469	2.6
WRPPH	674.3594	674.3527	6.7
WRPPH -28	646.3604	646.3578	2.6
RPPH	488.2748	488.2734	1.4
HVP/PHV	344.1884	344.188	0.4
PPH	332.175	332.1723	2.7
RPP	351.2127	351.2145	1.8
WRP	440.2427	440.241	1.7
VP	197.1296	197.129	0.6
HV	237.1371	237.1352	1.9
PH	235.121	235.1195	1.5
PP	195.1153	195.1134	1.9
RP	254.1632	254.1617	1.5
